# A necroptosis-independent function of RIPK3 promotes immune dysfunction and prevents control of chronic LCMV infection

**DOI:** 10.1038/s41419-023-05635-0

**Published:** 2023-02-15

**Authors:** Simon P. Preston, Cody C. Allison, Jan Schaefer, William Clow, Stefanie M. Bader, Sophie Collard, Wasan O. Forsyth, Michelle P. Clark, Alexandra L. Garnham, Connie S. N. Li-Wai-Suen, Thanushi Peiris, Jack Teale, Liana Mackiewicz, Sophia Davidson, Marcel Doerflinger, Marc Pellegrini

**Affiliations:** 1 Walter and Eliza Hall Institute of Medical Research, Parkville, VIC Australia; 2 Department of Medical Biology, The University of Melbourne, Parkville, VIC Australia; 3 SYNthesis Research, Bio21 Institute, Parkville, VIC Australia

**Keywords:** Immune cell death, Viral infection

## Abstract

Necroptosis is a lytic and inflammatory form of cell death that is highly constrained to mitigate detrimental collateral tissue damage and impaired immunity. These constraints make it difficult to define the relevance of necroptosis in diseases such as chronic and persistent viral infections and within individual organ systems. The role of necroptotic signalling is further complicated because proteins essential to this pathway, such as receptor interacting protein kinase 3 (RIPK3) and mixed lineage kinase domain-like (MLKL), have been implicated in roles outside of necroptotic signalling. We sought to address this issue by individually defining the role of RIPK3 and MLKL in chronic lymphocytic choriomeningitis virus (LCMV) infection. We investigated if necroptosis contributes to the death of LCMV-specific CD8^+^ T cells or virally infected target cells during infection. We provide evidence showing that necroptosis was redundant in the pathogenesis of acute forms of LCMV (Armstrong strain) and the early stages of chronic (Docile strain) LCMV infection in vivo. The number of immune cells, their specificity and reactivity towards viral antigens and viral loads are not altered in the absence of either MLKL or RIPK3 during acute and during the early stages of chronic LCMV infection. However, we identified that RIPK3 promotes immune dysfunction and prevents control of infection at later stages of chronic LCMV disease. This was not phenocopied by the loss of MLKL indicating that the phenotype was driven by a necroptosis-independent function of RIPK3. We provide evidence that RIPK3 signaling evoked a dysregulated type 1 interferone response which we linked to an impaired antiviral immune response and abrogated clearance of chronic LCMV infection.

## Introduction

Receptor interacting protein kinase 3 (RIPK3) and mixed lineage kinase domain-like (MLKL) are essential molecular components of the necroptotic cell death pathway. This lytic form of cell death releases damage associated molecular patterns (DAMPs) that cause immune activation and inflammation [1]. Necroptosis is not a default cell death pathway and a set of uncommon and complex circumstances are required to enable necroptotic signalling. Although easily studied in vitro, the role of necroptosis in disease pathogenesis at the level of a whole organism is difficult to define.

Tumour necrosis factor (TNF) is the most well characterised trigger for necroptosis [2]. Other death receptors such as Fas and TNF-related apoptosis inducing ligand (TRAIL) have also been described to trigger necroptosis in various inflammatory settings [3, 4]. The levels and stoichiometry of several key signalling molecules need to be perturbed, including dysregulation of inhibitor of apoptosis proteins (IAPs), caspase-8 and FLICE inhibitory protein (cFLIP), together with the provision of ligand/receptor signal (eg TNF/TNFR1), for necroptosis to be initiated [1, 2]. It is not clear under which physiological conditions such a sequence of events would occur to promote necroptosis. Certainly in chronic active infections, innate and adaptive immune cells contribute to the persistent production of TNF and FasL (at least membrane bound). Therefore, it would be important to understand if in these conditions, necroptosis could be contributing to disease pathogenesis.

Several mouse and human viruses encode proteins that inhibit apoptosis and / or necroptosis [5]. However, it is unclear if the viral antagonists of caspase-8 and RIPK3 produce partial loss of function across all or just some of the multiple signalling pathways that RIPK3 and caspase-8 are involved in. The confusion in the field is amplified by in vitro studies that lack context and physiological relevance. Lymphocytic choriomeningitis virus (LCMV) does not encode any proteins that inhibit caspase-8 and it does not directly cause lysis or death of infected cells and therefore by definition it cannot be a direct inducer of necroptosis. However, the virus does express pathogen associated molecular patterns (PAMPs) that may lead to RIPK3-mediated toll-like receptor (TLR) signaling, inflammasome activation, pyroptosis and inflammatory sequelae [6, 7]. Infection of mice with their natural pathogen, LCMV, may provide opportunity to examine RIPK3’s role outside of its necroptotic function. We cannot completely exclude the possibility that the inflammatory milieu and hyperactivated state during chronic LCMV infection does not indirectly sensitise cells to necroptosis, however this pathway can only be activated if the function of capase-8 is perturbed [1, 8, 9]. The protein levels of many signalling molecules dramatically change in immune cells upon activation so it is conceivable that immune cells, including hyperactivated T cells, may be prone to necroptosis in the absence of RIPK3 during chronic LCMV infection.

It is interesting to note that arguments supporting the significance of necroptosis as a means to combat infectious disease are primarily based on the importance of RIPK3 over and above MLKL [10–13]. This may well be attributed to RIPK3’s ability to mediate not only necroptosis but inflammation that contributes to pathogen control. Indeed, a recent study showed that RIPK3-dependent but MLKL-independent mechanisms protected against neuro-invasive west nile virus infection in mice [14]. RIPK3 was shown to be essential for cytokine and chemokine initiated immune control of infection. A number of other recent studies have described necroptotic and diverse non-necroptotic functions of RIPK3 [15, 16]. Consistent with this notion, perhaps the evolution of RIPK3 viral inhibitors was not to prevent necroptosis but rather to prevent RIPK3’s other roles in immune sigaling.

It is possible that necroptosis is neither advantageous to host or pathogen and that the evolution of viral inhibitors of RIPK3 have arisen to offset the consequences of caspase-8 inhibition and / or to prevent RIPK3’s functions outside of necroptosis. We were interested to understand if in hyperactivated T cells, that are repetitively stimulated by antigen and produce TNF, caspase-8 can prevent the induction of necroptosis. Our study aimed to investigate if necroptosis contributes to the death of LCMV-specific CD8^+^ T cells, or virally infected target cells during chronic LCMV infection. Importantly, we dissected the non-necroptotic roles of RIPK3, during the course of overwhelming LCMV infection, by directly comparing phenotypes and outcomes in RIPK3 and MLKL-deficient mice.

## Materials And methods

### Mice and LCMV infection and quantitation

Mice used in experiments were aged between 6–12 weeks. Gene-targeted animals used in experiments were all on a C57BL/6 (H-2D^b^) background and have been described elsewhere [17, 18]. All experiments were approved by the Walter and Eliza Hall Institute animal ethics committee. For all experiments, samples size was chosen to ensure adequate power (0.8). Mice were infected with 1 × 10^3^ pfu LCMV Armstrong or 2 × 10^6^ pfu LCMV docile by intravenous injection into the tail vein. LCMV docile was propagated on L929 cells (ATCC #CCL-1). Viral loads from organs of LCMV infected mice were determined by focus forming assay, using MC57G fibrosarcoma cells (ATCC #CRL-2295), as previously described [19].

### Flow cytometry

1 × 10^6^ splenocytes were taken for antibody staining and downstream analysis. Specific monoclonal antibodies were purchased from eBioscience (Thermofisher Scientific, Waltham, MA, USA) or BD Biosciences (San Jose, CA, USA): CD4 APC-Cy7 (RM4-5), CD8 BV510 (53-6.7), CD11b BV510 (M170), CD11c PECy7 (HL3), CD19 PerCP-Cy5.5 (ID3), CD69 BV421 (H1.2F3), CD279 PE (PD1; J43), Gr-1 Alexa700 (Ly6G/Ly6C; RB6-8C5), MHC-II FITC (I-A/I-E; 2G9) and CD16/32 (2.4G2). H-2D^b^ restricted LCMV tetramer staining and peptide restimulation were performed as previously described [20]. All flow cytometry data were collected on a Fortessa X20 or Fortessa1 (BD Biosciences) and analyzed using FlowJo software (version 10, Flowjo LLC, Ashland, OR, USA).

### In vitro macrophages assays

Thioglycollate medium (Sigma-Aldrich, St. Louis, MO, USA) was prepared in distilled water by boiling and then sterilised by autoclaving. Medium was then allowed to sit for >2 months. 5 days before isolating macrophages, 2 mL of thioglycollate medium was injected intraperitoneally into mice. To isolate macrophages, mice were euthanized and the peritoneal cavity flushed twice with 7 mL of ice-cold phosphate buffered saline (PBS). Macrophages were transferred into a 50 mL falcon tube before centrifugation at 500 x g for 5 min at 4 °C. Macrophages were resuspended in IMDM supplemented with 10 % FCS and transferred to a 24-well plate with 5 ×10^5^ cells per well. After allowing cells to attach by incubating the plate at 37 °C for 1 h, macrophages were left uninfected or were infected with LCMV docile at an MOI of 1. Macrophages were isolated following 24 h incubation at 37 °C by cell scraping to prepare for flow cytometry analysis. To determine cell viability, macrophages were incubated with propidium iodide immediately before analysis. Alternatively, macrophages were fixed by incubating cells in 2% formalin (Sigma-Aldrich) for 10 min at RT. Cells were permeabilised using saponin containing Fix/Perm kit (BD). LCMV infected macrophages were identified by incubating cells with VL4 antibody (1:100) for 1 h at 4 °C, before addition of an anti-rat-APC conjugated secondary antibody (1:10 000, BD). Cells were acquired by flow cytometry and analyzed as above.

### RNA sequencing

LCMV-specific CD8^+^ T cells were isolated from the spleen and pooled lymph nodes of mice 14 days post infection with LCMV docile by fluorescent-activated cell sorting (FACS). H-2D^b^ restricted LCMV tetramers recognizing the GP33 and NP396 epitopes were utilized for staining, following the same protocol as described previously [20]. RNA extraction was performed using the RNeasy® Mini Kit (Qiagen, Venlo, Netherlands). An input of <500 pg of total RNA were prepared and indexed separately for illumina sequencing using the SMART-seq^®^ v4 Ultra^®^ low input RNA kit (Clontech, Takara-Bio, Shiga, Japan) as per manufacturer’s instructions. Each library was quantified using the Agilent Tapestation and the Qubit™ DNA BR assay kit for Qubit 3.0® Fluorometer (Thermo Fisher Scientific). The indexed libraries were pooled and diluted to 1.5 pM for paired end sequencing (2 × 81 cycles) on a NextSeq 500 instrument using the v2 150 cycle High Output kit (illumina) as per manufacturer’s instructions. The base calling and quality scoring were determined using Real-Time Analysis on board software v2.4.6, while the FASTQ file generation and de-multiplexing utilised bcl2fastq conversion software v2.15.0.4.

The samples were sequenced using paired-end reads. All samples were aligned to the mm10 build of the mouse genome using the STAR [21] aligner (v 2.5.3a). In all cases, at least 88% of fragments (read pairs) mapped to the genome. Following alignment, all fragments overlapping mouse genes were summarized into counts using the featureCounts function of the Rsubread [22] software package (v 1.32.4). Genes were identified using Gencode annotation to the mm10 genome (v m23). At least 55% percent of fragments were assigned to genes for all samples. Differential expression analyses were then undertaken using the limma [23] (v 3.46.0) and edgeR [24] (v 3.32.1) software packages.

Prior to analysis all gender specific genes – Xist and those unique to the Y-chromosome; were removed to avoid gender biases. All non-protein coding immunoglobulin genes, ribosomal RNAs (rRNAs), T cell receptor genes, pseudo genes, and unknown genes were also removed. Expression based filtering was then performed using edgeR’s filterByExpr function with the minimum count set to 20. A total of 15 649 genes remained for downstream analysis. Compositional differences between the samples were then normalized using the trimmed mean of *M*-values (TMM) method [25]. Following filtering and normalization, counts were transformed to log_2_ counts per million (CPM) with associated observation level precision weights and sample weights using voom [26]. Differential expression between KO and WT samples was then assessed using linear models and robust empirical bayes moderated *t*-statistics (robust limma-voom pipeline). To increase precision, the linear models incorporated a factor representing experiment batch. The Benjamini and Hochberg method was used to control the false discovery rate (FDR) below 10%.

Pathway analyses of the Gene Ontology (GO) and Kyoto Encyclopedia of Genes and Genomes (KEGG) were conducted using limma’s goana and kegga functions respectively. Analysis of the Molecular Signatures Database Hallmark gene sets was achieved using limma’s fry gene set test.

The mean-difference plot and barcode plots were generated using limma’s plotMD and barcodeplot functions respectively. The heatmap was created using the pheatmap software package.

### Measurement of cytokines

The mouse IFNα platinum ELISA kit (normal sensitivity; Affymetrix eBioscience, Vienna, Austria) or the Verikine-HS mouse IFNα All subtype ELISA kit (high sensitivity; Pbl Assay Science, Piscataway, NJ, USA) was used to measure serum IFNα concentrations from LCMV infected mice. The Verikine mouse IFNβ ELISA kit (normal sensitivity; Pbl Assay Science) or the Verikine-HS mouse IFNβ ELISA kit (high sensitivity; Pbl Assay Science) was used to measure serum IFNβ concentrations from LCMV infected mice. These are standard ELISA kits that were performed according to manufacturer’s instructions. Alternatively, mouse IFNα and IFNβ were also measured using the mouse interferon dual plex (#EPX02A-22187-901, Thermofisher Scientific). A cytokine multiplex array was also performed using the ProcartaPlex™ 26-Plex Panel 1 (EPX01A-26088-901), according to manufacturer’s instructions (ProcartaPlex, Thermofisher Scientific). Plates were read using a Bioplex 200 analyser (Biorad, Hercules, CA, USA).

### qRT-PCR analysis of IFN-I and downstream targets

Livers from LCMV docile infected mice were harvested at 14 day post infection, 2 x 5 mm^2^ pieces were dissected from the right lobe and snap frozen in liquid nitrogen in 2 mL eppendorf and stored at −80 °C. Liver pieces were lysed using a tissue lyser (TisssueLyser II, Quiagen, 30/s, 6 min) in 1 ml of TRIzol (Invitrogen, #15596026). RNA was isolated as per manufacture’s instructions. Deoxyribonuclease (DNase)–treated total RNA (5 μg) was reverse-transcribed using oligo(dT) nucleotides (Promega) to generate complementary DNA (cDNA). qPCR was performed using SYBR Green/ROX qPCR Master Mix (Thermo Fisher Scientific) on a QuantStudio 12 K (Thermo Fisher Scientific). Gene expression was normalized against the expression of the housekeeping gene, *Gapdh*. Mouse specific primer sequences used are indicated in supplementary Table 1 (see supplementary material).

### Statistical Analysis

Prism 6.0d software (Graph Pad Software) was used to perform the statistical tests as indicated in the respective figure legends. Grouped data is represented as mean SD or mean SEM as indicated, error bars represent SD or SEM. ns *P* > 0.05 – not statistically significant. *P < 0.05, **P < 0.005, ***P < 0.001, ****P < 0.0001 – statistically significant.

## Results

### Loss of necroptotic mediators, RIPK3 or MLKL, does not alter immunity to acute LCMV infection

**Fig. 1:**
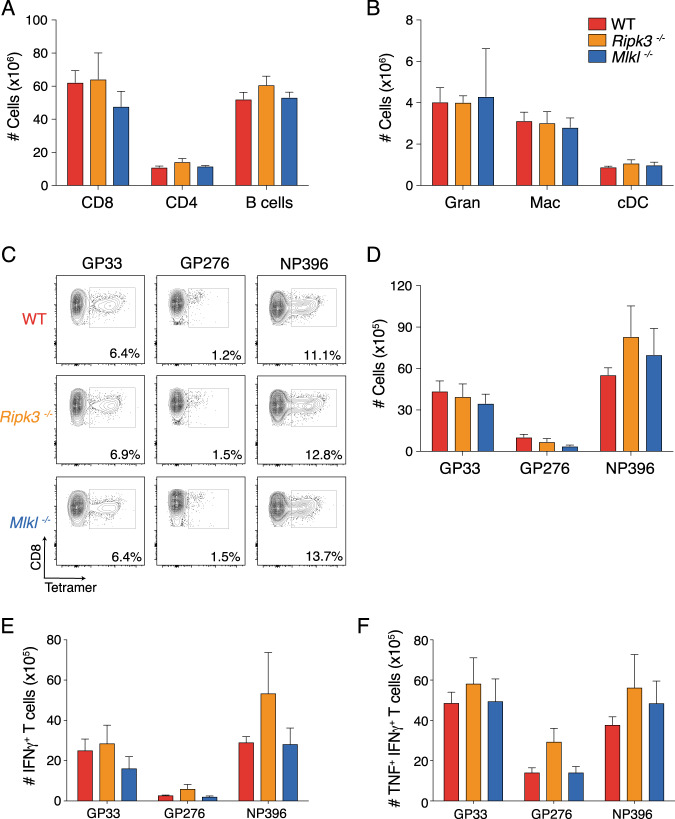
Necroptosis does not contribute to the control of acute LCMV infection. Total numbers of (**A**) lymphocytes and (**B**) myeloid cells isolated from the spleens of mice with the indicated genotypes (*n* = 4 mice per genotype) at 8 days post-infection with acute LCMV (Armstrong). (**C**) Representative flow cytometry plots showing the proportion of splenic CD8^+^ T cells that recognize LCMV-specific epitopes. **D** Total numbers of CD8^+^ T cells, with the indicated specificities, same experiment as (**C**) (*n* = 4 mice per genotype). **E**, **F** Ex vivo cytokine production by LCMV-specific CD8^+^ T cells. Total splenocytes were re-stimulated with the indicated recombinant cognate LCMV peptides. Numbers of CD8^+^ T cells producing the indicated cytokines (**E**, **F**) after restimulation (*n* = 4 mice per genotype). All data were obtained from mice at 8 days post infection with LCMV Armstrong. Mean and SEM are represented in bar graphs. Data in (**A**, **B**, **D**–**F**) are representative of two independent experiments. Flow cytometry plots (**C**) are representative of 8 analyses performed on independent mice.

To understand if necroptosis plays role in acute LCMV infection and disease, we infected WT, RIPK3-deficient (*Ripk3*^*−/−*^) or MLKL-deficient (*Mlkl*^*−/−*^) mice with a low dose LCMV Armstrong, an acute variant of the virus, and analyzed the immune response at day 8 post infection. Importantly, the steady state physiology of *Ripk3*^*−/−*^ and *Mlkl*^*−/−*^ mice have been thoroughly described and they appear identical to WT mice [17, 18]. We did not observe any differences in the total numbers of splenic CD8^+^ T cells, CD4^+^ T cells, B cells, granulocytes, inflammatory macrophages or conventional dendritic cells across infected mice regardless of genotype (Fig. 1A, B). There were no differences in the proportion or number of CD8^+^ T cells that specifically recognize the LCMV epitopes GP33, GP276 or NP396 across genotypes (Fig. 1C, D). LCMV Armstrong infection is completely cleared in WT mice within 7 days due to a robust CD8^+^ T cell response [20]. Beyond the three CD8^+^ T cell epitopes already quantified, we also identified CD8^+^ short lived effector cells (SLEC) and memory precursor effector cells (MPEC). This phenotyping enables the identification of an overall pool of short lived cytotoxic cells and long-lived memory precursor cells that recognize LCMV [27]. There were no differences observed in the proportions or numbers of either SLECs or MPECs between any of the groups of mice at 8 days post infection (Supplementary Fig. 1A, B). There were also no differences between groups when we characterised and compared the function of virus specific CD8^+^ T cells, measured by TNF and IFNγ production upon restimulation (Fig. 1E, F and Supplementary Fig. 1C). These data indicate that MLKL and RIPK3 do not impact immunity to and dynamics of acute LCMV infection. Therefore, necroptosis and any non-necroptotic roles of RIPK3 are redundant in host pathogen-dynamics, pathogenesis and immune control of acute LCMV infection.

### RIPK3 or MLKL deficiency does not alter the expansion or attrition of virus-specific CD8^+^ T cells during chronic LCMV infection

Infection with acute and chronic forms of LCMV have completely different host-pathogen dynamics and consequently different outcomes. C57BL/6 mice infected with chronic LCMV docile are unable to control infection. The protracted course of the disease involves the turnover of many generations of immune cell populations and allows long term study of factors that affect the complex and prolonged natural course of immunity to such pathogens. In order to examine the role of necroptosis on CD8^+^ T cell number and function during chronic LCMV infection, we infected RIPK3 and MLKL-deficient animals and performed analyses at days 8 and 35 post infection.

**Fig. 2:**
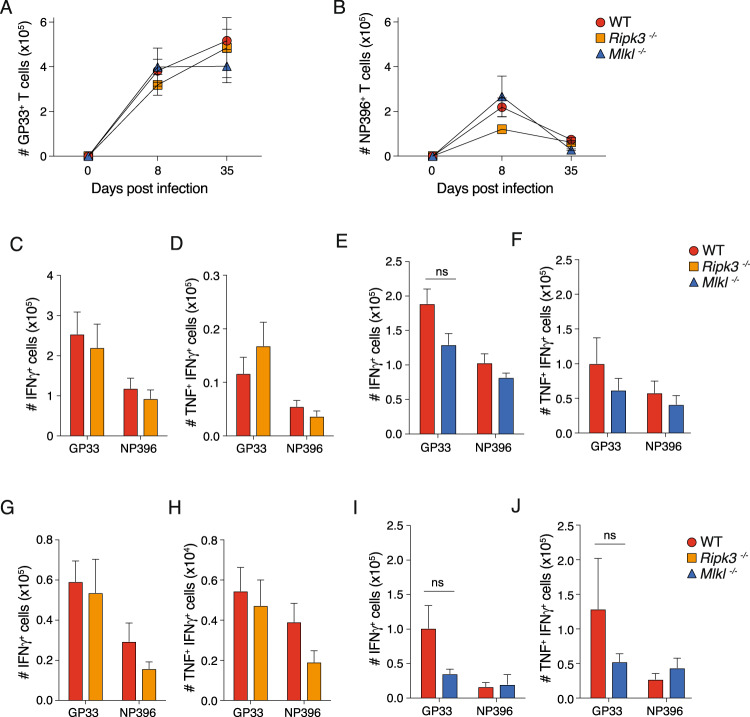
Loss of RIPK3 or MLKL does not alter the expansion or attrition of virus-specific CD8^+^ T cells during onset of chronic LCMV infection. **A**–**B** Absolute numbers of CD8^+^ T cells that specifically recognise the LCMV epitopes (**A**) GP33 or (**B**) NP396 from the spleens of mice, with the indicated genotypes, over the course of LCMV docile infection. **C**–**F** Ex vivo cytokine production by LCMV specific CD8^+^ T cells after restimulation with the indicated recombinant cognate LCMV peptides GP33 or NP396. CD8^+^ T cells were harvested from spleens of mice with the indicated genotypes at 8 days post infection with LCMV docile. **G**–**J** Ex vivo cytokine production as in (**C**–**F**), 35 days post infection. All data are combined from two independent experiments. Graphs showing summary data indicate mean and SEM. NS, not significant *p* > 0.05 (unpaired *t* test).

The loss of RIPK3 or MLKL had no impact on the expansion or attrition of LCMV specific T cells as evidenced by comparable kinetics in virus-specific CD8^+^ T cells that recognize GP33 and NP396 across both RIPK3 and MLKL-deficient mice compared to WT mice at 8- and 35-days post infection (Fig. 2A, B). Chronic LCMV infection causes the loss of high affinity, virus-specific CD8^+^ T cells that are capable of producing both TNF and IFNγ [28]. Using ex vivo peptide re-stimulation, there was a comparable loss of cytokine producing virus-specific CD8^+^ T across *Ripk3*^*−/−*^, *Mlkl*^*−/−*^ and WT animals at both day 8- and 35-post infection (Fig. 2C–J and Supplementary Fig. 2A–D).

### Loss of RIPK3 but not MLKL promotes the clearance of LCMV at latter stages of chronic infection

**Fig. 3:**
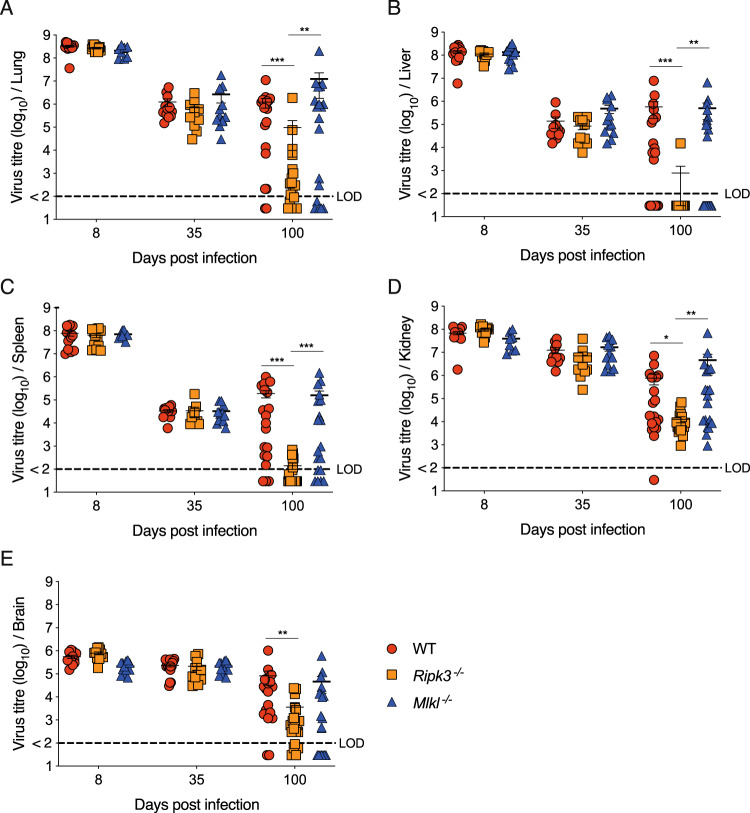
Non-necroptotic RIPK3 signaling prevents clearance of chronic LCMV at late stages of infection. Virus titres in mice with the indicated genotypes, across various organs at indicated timepoints postinfection with LCMV docile. Viral loads were titred in the **A** lung, **B** liver, **C** spleen, **D** kidney and **E** brain. Each symbol represents one mouse. Dotted line indicates the reliable limit of detection (LOD) for the assay. The median is indicated by a horizontal line in each group at each time point. *p < 0.05, **p < 0.01, ***p < 0.005 (one-way ANOVA followed by Dunnett’s multiple comparisons test).

To determine and compare the effect of RIPK3 or MLKL-deficiency during late stages of LCMV docile infection, we measured the virals loads in the lung, liver, spleen, kidney and brain at 8, 35 and 100 days post infection. There were no differences in viral titres during the initial and early (day 8 and 35) phases of chronic infection. However, in the late persistent stage at 100 days post-infection *Ripk3*^*−/−*^ mice exhibited enhanced viral clearance kinetics across multiple organs in contrast to poor viral control in WT and *Mlkl*^*−/−*^ infected mice (Fig. 3A–E). These data support the notion that non-necroptotic functions of RIPK3 dampen the host’s ability to respond to persistent LCMV infection.

### Loss of RIPK3 doesn’t alter the function of virus-specific T cells during chronic infection

In order to identify the underlying cause of enhanced viral clearance in RIPK3-deficient animals at late stages of persistent infection, we thoroughly investigated the immune cell compartment of *Ripk3*^*−/−*^ mice compared to WT mice throughout the entire course of chronic LCMV infection. Immune cell subset phenotyping showed that there were no differences observed in CD8^+^ T cells, CD4^+^ T cells or macrophages between groups at any of the time points investigated (Supplementary Fig. 3A–C). Intrestingly, there was a small but transient increase in the numbers of granulocytes, cDCs and B cells in *Ripk3*^*−/−*^ mice compared to WT, within the first week post infection (Supplementary Fig. 3D–F).

**Fig. 4:**
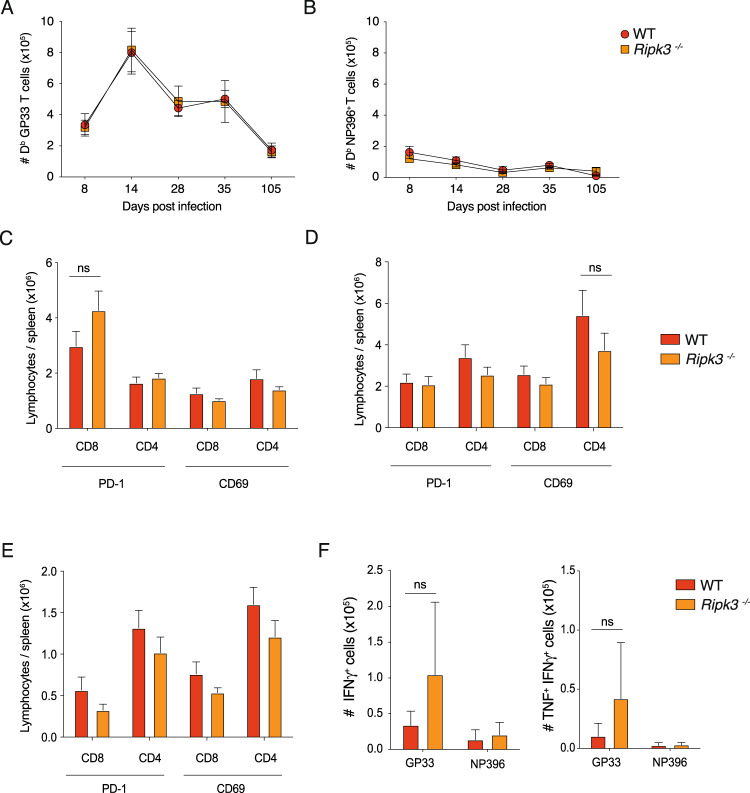
Loss of RIPK3 does not alter the numbers and activation status of CD8^+^ T cells responding to infection throughout chronic LCMV infection. **A**, **B** Absolute numbers of CD8^+^ T cells that specifically recognise the LCMV epitopes (**A**) GP33 or (**B**) NP396 from the spleens of WT or RIPK3-deficient mice over the course of LCMV docile infection (*n* = 8-12). **C** Absolute numbers CD4 or CD8 cells that express PD-1 or CD69 from the spleens of WT or RIPK3-deficient mice 8 days pos-infection with LCMV docile infection (*n* = 4). **D** Absolute numbers CD4 or CD8 cells that express PD-1 or CD69 from the spleens of WT or RIPK3-deficient mice 35 days post-infection with LCMV docile infection (*n* = 4 per genotype). **E** Absolute numbers CD4 or CD8 cells that express PD-1 or CD69 from the spleens of WT or RIPK3-deficient mice 105 days post-infection with LCMV docile infection (*n* = 4 per genotype). **F** Average numbers of cytokine producing CD8^+^ T cells from the spleens of WT or RIPK3-deficient mice 105 days post-infection with LCMV docile infection.after ex vivo restimulation with the indicated peptides (*n* = 4 per genotype). Data in **A**, **B** and **C**–**F** were combined data from 10 and 2 experiments, respectively. Graphs showing summary data indicate mean and SEM. NS not significant *p* > 0.005 (unpaired *t* test).

We showed that the absolute numbers of CD8^+^ T cells that specifically recognize LCMV epitopes GP33 or NP396 from the spleens of infected mice did not differ between *Ripk3*^*−/−*^ and WT animals during onset of chronic infection at days 8 and 35 (Fig. 2A–B). We obtained similar results at intermediate timepoints of infection (days 14 and 28), as well as late stages of persistent infection at day 105 (Fig. 4A, B). In the context of chronic LCMV, CD69 and PD-1 expression on CD8^+^ and CD4^+^ T cells are markers of TCR-mediated acute and chronic activation/stimulation, respectively [29]. These markers enable the identification of the larger pool of LCMV-specific CD4^+^ and CD8^+^ T cells that are responding to the infection. There were no differences observed in the number or proportion of CD69^+^ or PD-1^+^ T cells in the spleens of WT compared to *Ripk3*^*−/−*^ mice at day 8, 35 or 105 post infection (Fig. 4C–E). Furthermore, no difference was detected between between *Ripk3*^*−/−*^ and WT animals when analysing high affinity, virus-specific CD8^+^ T cells capable of producing both TNF and IFNγ using ex vivo peptide re-stimulation (Fig. 4F).

Given that there was enhanced control of LCMV in *Ripk3*^*−/−*^ mice, we sought to exclude the possibility that the virus was not able to infect/propagate in RIPK3-deficient cells due to some intrinsic mechanism. Chronic strains of LCMV, including docile, can infect almost any cell in the mouse but it preferentially replicates within myeloid cells [30]. We isolated thioglycolate-illicited primary macrophages from uninfected animals and found that *Ripk3*^*−/−*^ macrophages were equally susceptible to LCMV infection compared to WT macrophages following 24 h of ex vivo infection (Supplementary Fig. 4A). Although not directly examined, a replicative defect causing a quantitative drop in virus production in *Ripk3*^*−/−*^ cells is an unlikely explanation for the observed differences in viral loads because this defect would have manifested as reduction in viraemia at early time points. We also assessed the viability of LCMV infected macrophages and did not detect any differences in viability between WT and *Ripk3*^*−/−*^ cells (Supplementary Fig. 4B).

### RNA sequencing revealed perturbations in interferon signaling pathways in *Ripk3*^*-/-*^ virus-specific CD8^+^ T cells

**Fig. 5:**
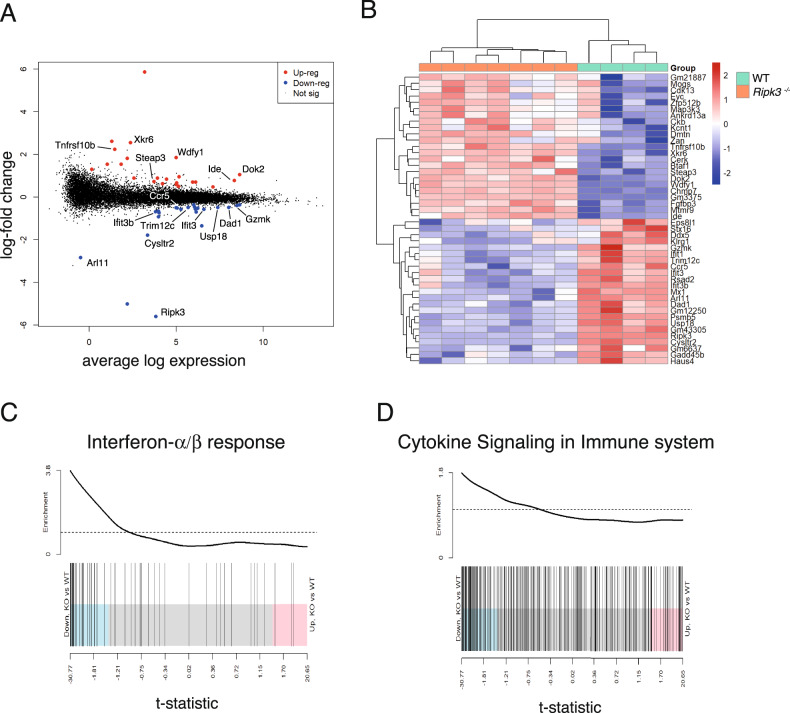
RNA sequencing reveals a depressed interferon signature in RIPK3-deficient mice infected with LCMV docile. **A** MD plot showing the genes downregulated (blue) or upregulated (red) in *Ripk3*^*−/−*^ LCMV-specific CD8^+^ T cells versus WT. Genes of interest are indicated. **B** Heatmap of 46 significantly differentially expressed genes (*p* < 0.05) in LCMV-specific CD8^+^ T cells from WT mice compared to *Ripk3*^−/−^ mice (mean of *n* = 4 WT, *n* = 7 *Ripk3*^*−/−*^ per group, differential expression assessed u sing a robust empirical bayes procedure, FDR controlled below 0.10 using Benjamini and Hochburg method). Colour code indicates Z-score. **C** Barcode plot analyses of Hallmark gene set analysis for genes involved in interferon-α/β response in LCMV-specific CD8^+^ T cells from *Ripk3*^*−/−*^ mice compared to WT mice (gene enrichment test using ROAST method). **D** Barcode plot analyses of curated gene set of genes involved in interferon-α/β response in LCMV-specific CD8^+^ T cells from *Ripk3*^*−/−*^ mice compared to WT mice (gene enrichment test using ROAST method). All data in was obtained from flow-cytometry sorted T cells, isolated from animals sacrificed 14 days post infection with LCMV docile.

We attempted to identify a potential mechanism underlying the differential clearance kinetic in RIPK3-deficient animals, and performed unbiased RNAseq analysis of FACS-sorted virus-specific CD8^+^ T cells from WT and *Ripk3*^*−/−*^ mice at 14-days post-infection with LCMV docile. A total of 46 genes were identified to be differentially expressed (DE) between WT and *Ripk3*^*−/−*^ CD8^+^ T cells at this time point (Fig. 5A–B). Pathway analysis revealed dysregulation in cytokine signaling and a depressed type I interferon (IFN-I) response and a downregulation in interferon signaling in CD8^+^ T cells from *Ripk3*^*−/−*^ mice compared to WT mice (Fig. 5C, D).

### RIPK3 driven Type 1 interferon signalling constrains immunity to chronic LCMV infection

**Fig. 6:**
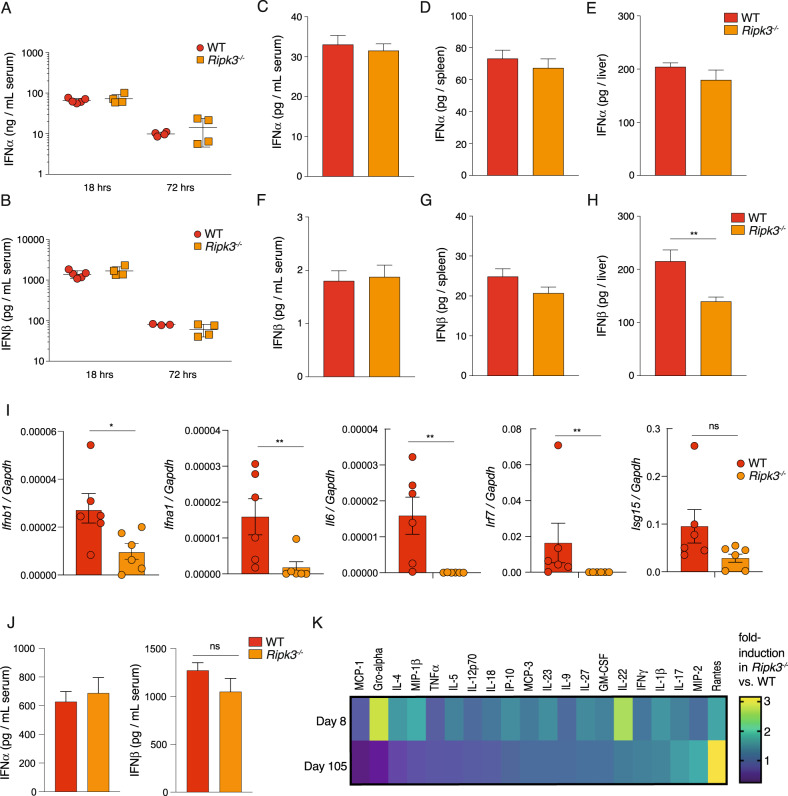
Dampened IFN-I levels in livers and overall changes in circulating cytokines detected in Ripk3^-/-^ mice during chronic LCMV infection. Serum (**A**) IFNα or (**B**) IFNβ levels were quantified by ELISA, 18 h or 72 h after LCMV docile infection. **C**–**E** IFNα or (**F**–**H**) IFNβ levels were quantified in the serum and indicated organs 8 days post LCMV docile infection. (**C**–**H**) represent 8–9 mice per group. The mean and SEM are indicated. **p < 0.01 (unpaired *t* test). HRS hours. **I** q-RT PCR gene expression analysis (relative to *Gapdh*) of *Ifnb1, Ifna1, Il6, Irf7* and *Isg15* in whole livers from WT and RIPK3-deficient animals at 14 days post LCMV docile infection. The mean and SEM are indicated, **p < 0.01, *p < 0.05, ns > 0.05 (Mann–Whitney *U* test, two-tailed). (*n* = 6 mice per group). **J** IFNα or IFNβ levels in serum of WT or *Ripk3*^−/−^ mice at 105 days post infection with LCMV docile, quantified by dual plex ELISA (per group: *n* = 6 WT, *n* = 6 *Ripk3*^−/−^). **K** Multiplex cytokine array was performed on sera collected from LCMV docile infected mice at 8 days and 105 days post infection. Heatmap represents average fold-change between WT and RIPK3-deficient mice at indicated time points (*n* = 4 mice per genotype).

Type I interferons (IFN-I) are best recognized for their multifaceted anti-viral effects during acute infections. It has become apparent, however, that persistent IFN-I signalling can be detrimental to many aspects of immunity during chronic persistent infections [31]. Early blockade of IFN-I signalling does not alter the immediate number of LCMV-specific CD8^+^ T cells but does augment enhanced control during the later stages of chronic infection [32, 33]. RIPK3 has been implicated in promoting IFN-I responses across a diverse range of viral and bacterial pathogens [34, 35]. This activity is dependent on RIPK3’s kinase function and likely involves interaction with mitochondrial antiviral-signalling protein (MAVS). We quantified serum levels of IFNα and IFNβ at several time points. WT and *Ripk3*^*−/−*^ mice infected with LCMV docile had similar levels of IFN-I during the early IFN-I burst (18–72 h; Fig. 6A, B). At day 8 post infection, we detected a significant decrease in IFNβ levels but only slight decrease in IFNα, in the livers of *Ripk3*^*−/−*^ mice compared to control animals. No other differences were observed in the serum or spleen (Fig. 6C–H). We confirmed these findings and performed qPCR analysis to quantify the gene expression levels of IFN-I as well as downstream Interferon Stimulated Genes (ISGs) in livers of WT and *Ripk3*^*−/−*^ mice. Both *Ifna1* and *Ifnb1*, as well as the downstram mediators *Il6*, *Irf7* and *Isg15* were expressed at significantly lower levels in RIPK3-deficient animals (Fig. 6I). Aditionally, we analysed the blood cytokine profiles both at onset of infection (day 8) as well as at late stages of infection (105 days), when marked differences in viral clearance kinetics manifest. Similar to day 8, no striking difference was detected in serum IFNα and in IFNβ at late stage infection (day 105) (Fig. 6J). However, we observed overall changes in the global pattern of circulating serum cytokines in *Ripk3*^*−/−*^ mice compared to control animals (Fig. 6K). This included key cytokines involved in regulating antiviral responses such as IL-22 [36], IL-23 [37] and IL-27 [38, 39] (Fig. 6K).

## Discussion

The significance of necroptosis as a host defence mechanism against infection has been primarily defined by studies investigating the role of RIPK3 rather than MLKL. However, recent evidence suggest that RIPK3’s ability to mediate inflammation may contribute to pathogen control over and above its role as initiator of necroptotic cell death [14]. A range of diverse non-necroptotic functions have been attributed to RIPK3, including a central role in cytokine and chemokine initiated immune control of infection, and RIPK3-dependent but MLKL-independent mechanisms have been implicated in immune defense [14–16].

The present study aimed to clarify the role of RIPK3 and MLKL during acute and chronic LCMV infection. Our data indicates that necroptosis is redundant in the pathogenesis of both acute and chronic LCMV infection, as loss of MLKL had no impact on the adaptive immune response to LCMV and no differences were detected in viral loads across several tissues during the early, adaptive and persistant stages of chronic infection. Similarly, RIPK3 deficiency had no impact on adaptive immunity and viral loads in the early and intermediate phases of chronic infection. However, as *Ripk3*^*−/−*^ mice had significantly lower viral loads at late persistant stages of chronic LCMV infection, we hypothesized that RIPK3 promotes immune dysfunction and prevents viral clearance by a necroptosis-independent function. While pleiotropic functions for RIPK3 in the restriction of viral pathogenesis in acute viral infection have been demonstrated in the context of WNV [14], there are no reports presently in the literature describing non-necroptotic functions of RIPK3 in chronic persistent infection. In order to identify a potential mechanism underlying the differential clearance kinetic in RIPK3-deficient animals, we performed unbiased bulk RNAseq analysis of FACS-sorted LCMV-specific CD8 + T cells which revealed perturbed IFN-I signaling.

Based on these results and reports of the potential role for RIPK3 in enhancing IFN-I signalling during acute infections [34, 35], we speculated that RIPK3 may play a role in maintaining detrimentally high levels of this cytokine during the course of chronic LCMV infection. Although the serum levels of IFN-Is are very high in the first 1–2 days following infection, it is almost undetectable throughout the remaining course of infection. We observed significant differences in IFNβ but not IFNα protein levels in the liver at day 8 post infection, and no differences in spleen or serum IFNβ or IFNα in *Ripk3*^*−/−*^ mice compared to WT counterparts. The reduction in LCMV viral titres in the liver of RIPK3 mice at later stages during chronic infection was more pronounced compared to other organs. Although RIPK3 protein levels are transcriptionally, and consequently functionally, repressed in hepatocytes [40], RIPK3 is highly expressed in Kupffer cells [40]. Although some level of type I IFN is required to control acute forms of LCMV [41] excessive or prolonged production of Type I IFN may affect hepatocyte metabolism leading to altered T Cell function [42]. Therefore, we speculate that a gain of function in adaptive immunity will be most evident, even at early time points in the liver. Additionally, the observation that differences in IFNβ and not IFNα contributes to the control of chronic infection is consistent with previously published work that utilised IFN-I antibody blockade to enhance CD8^+^ effector function in chronic LCMV [43]. Consistent with the notion that RIPK3-mediated signalling might evoke a dysregulated type 1 interferon response, we detected a change in the expression patterns of the Interferone Regulated Genes (ISGs) *Irf7*, *Il6* and *Isg15* early after infection (day 14), as well as in the pattern of circulating serum cytokines in RIPK3-deficient animals both at early (day 8) and later (day 105) time points. This indicated global alterations in the immune response during chronic infection with LCMV in the absence of RIPK3. Importantly, this included key cytokines involved in the regulation of antiviral responses such as IL-22 [36], IL-23 [37] and IL-27 [38, 39]. The source of these cytokines remains to be identified, however the increase in dendritic cells (DCs) we observed in RIPK3-deficient animals during the intermediate phase of infection might provide clues, given dendritic cells are also known to produce high amounts of type I IFN [44].

A conundrum with our data is that viral loads fall at the late stages of persistent infection in RIPK3-deficient mice compared to WT animals but the absolute number of effector immune cells capable of clearing infection is similar between genotypes at this time. Superficially this could be interpreted as non-immunologically mediated clearance of LCMV. However, we found no viral replicative defect or host cell death phenotype in primary RIPK3 deficient cells infected in vitro. The likely explanation for virological control, is that although the absolute number of virus speficic T cells remains constant across genotypes the decline in viral loads in infected RIPK3-deficient mice compared to controls translates to 2–3 log increase in the ratio of immune effector cells to virus. This profound alternation in the ratio of immune effector cells to viral loads is rarely seen in LCMV studies attempting to augment immunity and likely explains the clearance and elimination of chronic LCMV in RIPK3-deficient mice.

Collectively, our data provided evidence for a non-necroptotic function of RIPK3 in pathogen control and immune response during chronic viral infection. While we identify dysregulated type 1 interferon signaling as potential mechanism, a thorough analysis of IFN-I levels in RIPK3-deficient mice across numerous infections is warranted to help unravel the complexities caused by RIPK3’s multiple roles. Additionally, a thorough comparison of infection phenotypes across RIPK3 and MLKL-deficient mice is essential in all future studies attempting to attribute clinical outcomes to RIPK3 versus necroptosis. These insights have implications for the development of RIPK3 inhibitors that may have therapeutic roles in the treatment of chronic persistent infections.

## Supplementary information

Supplemental Material

checklist

## Data Availability

Generated RNA seq data will be deposited in public respository.

## References

[1] Newton K, Manning G (2016). Necroptosis and inflammation. Annu Rev Biochem.

[2] Pasparakis M, Vandenabeele P (2015). Necroptosis and its role in inflammation. Nature.

[3] He S, Wang X (2003). RIP kinases as modulators of inflammation and immunity. Nat Immunol.

[4] Peter ME, Krammer PH (2003). The CD95(APO-1/Fas) DISC and beyond. Cell death Differ.

[5] Upton JW, Shubina M, Balachandran S (2017). RIPK3-driven cell death during virus infections. Immunol Rev.

[6] Kang S, Fernandes-Alnemri T, Rogers K, Mayes L, Wang Y, Dillon C (2015). Caspase-8 scaffolding function and MLKL regulate NLRP3 inflammasome activation downstream of TLR3. Nat Commun.

[7] Wang X, Jiang W, Yan Y, Gong T, Han J, Tin Z (2014). RNA viruses promote activation of the NLRP3 inflammasome through a RIP1-RIP3-DRP1 signaling pathway. Nat Immunol.

[8] Silke J, Rickard JA, Gerlic M (2015). The diverse role of RIP kinases in necroptosis and inflammation. Nat Immunol.

[9] Grootjans S, Berghe TV, Vandenabeele P (2017). Initiation and execution mechanisms of necroptosis: an overview. Cell death Differ.

[10] Cho YS, Challa S, Moquin D, Genga R, Ray TG, Guildford M (2009). Phosphorylation-driven assembly of the RIP1-RIP3 complex regulates programmed necrosis and virus-induced inflammation. Cell.

[11] Upton JW, Kaiser WJ, Mocarski ES (2012). DAI/ZBP1/DLM-1 complexes with RIP3 to mediate virus-induced programmed necrosis that is targeted by murine cytomegalovirus vIRA. Cell Host Microbe.

[12] Pearson JS, Murphy JM (2017). Down the rabbit hole: is necroptosis truly an innate response to infection?. Cell Microbiol.

[13] Upton JW, Kaiser WJ, Mocarski ES (2010). Virus inhibition of RIP3-dependent necrosis. Cell Host Microbe.

[14] Daniels BP, Snyder AG, Olsen TA, Orozco S, Oguin TH, Tait SW (2017). RIPK3 restricts viral pathogenesis via cell death-independent neuroinflammation. Cell.

[15] Moriwaki K, Chan FKM (2017). The inflammatory signal adaptor RIPK3: functions beyond necroptosis. Int Rev Cell Mol Biol.

[16] Shlomovitz I, Zargrian S, Gerlic M (2017). Mechanisms of RIPK3-induced inflammation. Immunol Cell Biol.

[17] Murphy JM, Czabotar PE, Hildebrand JM, Lucet IS, Zhang JG, Alvarez-Diaz S (2013). The pseudokinase MLKL mediates necroptosis via a molecular switch mechanism. Immunity.

[18] Newton K, Sun X, Dixit VM (2004). Kinase RIP3 is dispensable for normal NF-kappa Bs signaling by the B-cell and T-cell receptors, tumor necrosis factor receptor, and Toll-like receptors 2 and 4. Mol Cell Biol.

[19] Battegay M, Cooper S, Althage A, Baenziger J, Hengartner H, Zinkernagel RM (1991). Quantification of lymphocytic choriomeningitis virus with an immunological focus assay in 24- or 96-well plates. J Virol Methods.

[20] Preston SP, Doerflinger M, Scott HW, Allison CC, Horton M, Cooney J (2021). The role of MKK4 in T‐cell development and immunity to viral infections. Immunol Cell Biol.

[21] Dobin A, Davis CA, Schlesinger F, Drenkow J, Zaleski C, Jha S (2013). STAR: ultrafast universal RNA-seq aligner. Bioinformatics.

[22] Liao Y, Smyth GK, Shi W (2019). The R package Rsubread is easier, faster, cheaper and better for alignment and quantification of RNA sequencing reads. Nucleic Acids Res.

[23] Ritchie ME, Phipson B, Wu D, Hu Y, Law CW, Shi W (2015). limma powers differential expression analyses for RNA-sequencing and microarray studies. Nucleic Acids Res.

[24] Robinson MD, McCarthy DJ, Smyth GK (2010). edgeR: a Bioconductor package for differential expression analysis of digital gene expression data. Bioinformatics.

[25] Robinson MD, Oshlack A (2010). A scaling normalization method for differential expression analysis of RNA-seq data. Genome Biol.

[26] Liu R, Holik AZ, Su S, Jansz N, Chen K, Leong HS (2015). Why weight? Modelling sample and observational level variability improves power in RNA-seq analyses. Nucleic Acids Res.

[27] Joshi NS, Cui W, Chandele A, Lee HK, Urso DR, Hagman J (2007). Inflammation directs memory precursor and short-lived effector CD8(+) T cell fates via the graded expression of T-bet transcription factor. Immunity.

[28] Pellegrini M, Calzascia T, Toe JG, Preston SP, Lin AE, Elford AR (2011). IL-7 engages multiple mechanisms to overcome chronic viral infection and limit organ pathology. Cell.

[29] Wherry EJ (2011). T cell exhaustion. Nat Immunol.

[30] Klepper A, Branch AD (2015). Macrophages and the viral dissemination super highway. EC Microbiol.

[31] Dagenais-Lussier X, Loucif H, Murira A, Laulhe X, Staeger S, Lamarre A (2017). Sustained IFN-I expression during established persistent viral infection: a “bad seed” for protective immunity. Viruses.

[32] Teijaro JR, Ng C, Lee AM, Sullivan BM, Sheehan KCF, Welch M (2013). Persistent LCMV infection is controlled by blockade of type I interferon signaling. Science.

[33] Wilson EB, Yamada DH, Elsaesser H, Herskovitz J, Deng J, Cheng G (2013). Blockade of chronic type I interferon signaling to control persistent LCMV infection. Science.

[34] Downey J, Pernet E, Coulombe F, Allard B, Meunier I, Jaworska J (2017). RIPK3 interacts with MAVS to regulate type I IFN-mediated immunity to Influenza A virus infection. PLoS Pathog.

[35] Saleh D, Najjar M, Zelic M, Shah S, Nogusa S, Polykratis A (2017). Kinase activities of RIPK1 and RIPK3 can direct IFN-β synthesis induced by lipopolysaccharide. J Immunol.

[36] Novelli F, Casenova JL (2004). The role of IL-12, IL-23 and IFN-γ in immunity to viruses. Cytokine Growth Factor Rev.

[37] Yi P, Liang Y, Ming Kee Yuan D, Jie Z, Kwota Z, Chen Y (2017). A tightly regulated IL-22 response maintains immune functions and homeostasis in systemic viral infection. Sci Rep.

[38] Huang Z, Zak J, Pratumchai I, Shaabani N, Vartabedian VF, Nguyen N (2019). IL-27 promotoes the expansion of self-renewing CD8+ T cells in persistant viral infection. J Exp Med.

[39] Harker JA, Wong KA, Dallari S, Bao P, Dolgoter A, Jo Y (2018). Interleukin-27R signaling mediates early viral containment and impacts innate and adaptive immunity after chronic lymphocytic choriomeningitis virus infection. J Virol.

[40] Preston SP, Stutz MD, Allison CC, Nachbur U, Gouil Q, Tran BM (2022). Epigenetic silencing of RIPK3 in hepatocytes prevents MLKL-mediated necroptosis from contributing to liver pathologies. Gastroenterology.

[41] Lang PA, Recher M, Honke N, Scheu S, Borkens S, Gailus N (2010). Tissue macrophages suppress viral replication and prevent severe immunopathology in an interferon-I-dependent manner in mice. Hepatology.

[42] Lercher A, Bhattacharya A, Popa AM, Caldera M, Schlapansky MF, Baazim H (2019). Type I interferon signaling disrupts the hepatic urea cycle and alters systemic metabolism to suppress T cell function. Immunity.

[43] Ng CT, Sullivan MB, Teijaro JR, Lee AM, Welch M, Rice S (2015). Blockade of interferon beta, but not interferon alpha, signaling controls persistent viral infection. Cell Host Microbe.

[44] Cella M, Jarrossay D, Facchetti F, Alebardi O, Nakajima H, Lanzavecchia A (1999). Plasmacytoid monocytes migrate to inflamed lymph nodes and produce large amounts of type I interferon. Nat Med.

