# Supplementary material for: A necroptosis-independent function of RIPK3 promotes immune dysfunction and prevents control of chronic LCMV infection
Source: Cell Death Dis. 2023 Feb 15;14(2):123. doi: 10.1038/s41419-023-05635-0 (PMC9931694; doi:10.1038/s41419-023-05635-0)
Supplement: Supplementary file 1 — Supplemental Material [file 41419_2023_5635_MOESM1_ESM.docx]

**Supplemental Information**

**A necroptosis-independent function of RIPK3 promotes immune dysfunction and prevents control of chronic LCMV infection**

Running title: RIPK3 abrogates control of chronic LCMV infection

Simon P Preston^1,2^, Cody C Allison^1,2^, Jan Schaefer^1,2^, William Clow^1,2^, Stefanie M Bader^1,2^, Sophie Collard^1,2^, Wasan O Forsyth^1,2^, Michelle P Clark^1,2^, Alexandra L Garnham^1,2^, Connie S N Li-Wai-Suen^1,2^, Thanushi Peiris^1^, Jack Teale^1^, Liana Mackiewicz^1^, Sophia Davidson^1,2^, Marcel Doerflinger^#,1,2^ and Marc Pellegrini^#,1,2^

^1^ Walter and Eliza Hall Institute of Medical Research, Parkville, Victoria, Australia

^2^ Department of Medical Biology, The University of Melbourne, Parkville, Victoria, Australia

**Supplementary Table 1: Oligonucleotide primers for qRT-PCR**

| *Ifnb1 fwd* | CCAGCTCCAAGAAAGGACGA |
| --- | --- |
| *Ifnb1 rev* | TGGATGGCAAAGGCAGTGTA |
| *Ifna1 fwd* | CTACTGGCCAACCTGCTCTC |
| *Ifna1 rev* | CCTTCTTGATCTGCTGGGCA |
| *Irf7 fwd* | AAGCTGGAGCCATGGGTATG |
| *Irf7 rev* | CGATGTCTTCGTAGAGACTGTTGG |
| *Il6 fwd* | CCAGAAACCGCTATGAAGTTCC |
| *Il6 rev* | CGGACTTGTGAAGTAGGGAAGG |
| *Isg15 fwd* | TGTGAGAGCAAGCAGCCAGA |
| *Isg15 rev* | CCCCCAGCATCTTCACCTTT |
| *Gapdh fwd* | CCAGGTTGTCTCCTGCGACTT |
| *Gapdh Rev* | CCTGTTGCTGTAGCCGTATTCA |

**
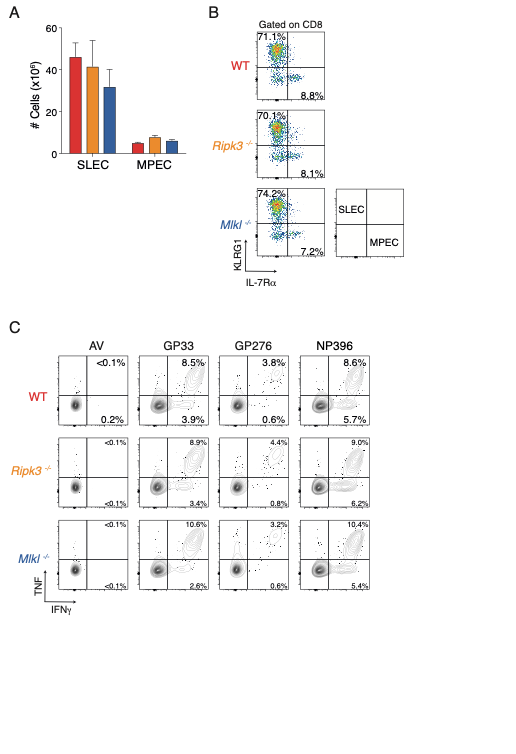
**

**Supplementary Figure 1.**

**Supplementary Figure 1.**

**(A)** Total SLECs and MPECs from the spleens of mice with the indicated genotypes (*n*=4) at 8 days post-infection with acute LCMV (Armstrong).

**(B)** Representative flow cytometry plots indicating the proportion of CD8+ T cells that express the SLEC or MPEC phenotype in mice with the indicated genotypes.

**(C)** Representative flow cytometry plots indicating the proportion of splenic CD8^+^ T cells producing cytokines following restimulation. Total splenocytes were re-stimulated with a control adenoviral (AV) peptide or the indicated recombinant cognate LCMV peptides. Proportions in quadrants are indicated.

All data were obtained from mice 8 days post infection with LCMV Armstrong. Mean and SEM are represented in bar graphs. Data in **(A)** are representative of two independent experiments. Flow cytometry plots **(B-C)** are representative of 8 analyses performed on independent mice. SLEC, short-lived effector cells (KLRG1^+^ IL-7Ra^-^); MPEC, memory precursor effector cell (KLRG1^-^ IL-7Ra^+^).

**
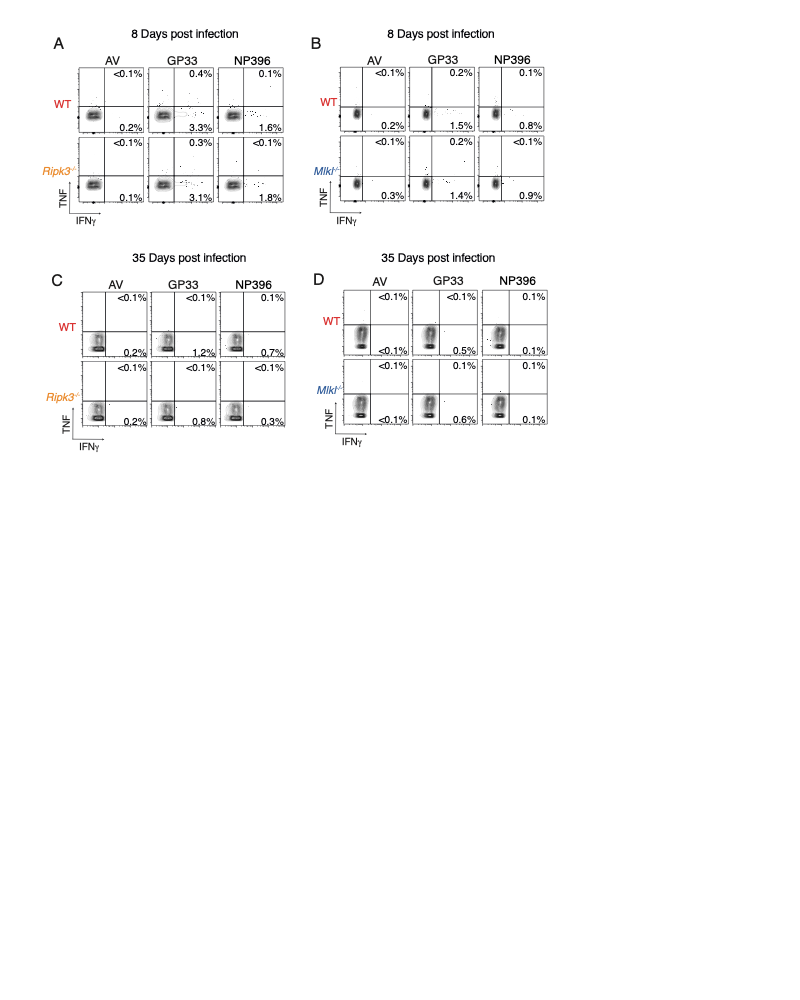
**

**Supplementary Figure 2.**

**Supplementary Figure 2.**

**(A+B)** Representative flow cytometry plots of analyses of *ex vivo* cytokine production by LCMV specific CD8^+^ T cells from spleens of mice with the indicated genotypes at 8 days post infection with proportions of cells recognizing LCMV epitopes GP33 or NP396 indicated in quadrants.

**(C+D)** Representative flow cytometry plots of analyses of *ex vivo* cytokine production by LCMV specific CD8^+^ T cells from spleens of mice with the indicated genotypes at 35 days post infection with proportions of cells recognizing LCMV epitopes GP33 or NP396 indicated in quadrants.

All Flow cytometry plots are representative of 8 analyses performed on independent mice.

**Supplementary Figure 3.**

**Supplementary Figure 3.**

Flow cytometric determination of the absolute number of

**(A)** CD8^+^ T cells, **(B)** CD4^+^ T cells, **(C)** Macrophages, **(D)** Granulocytes, **(E)** cDCs and **(F)** B cells isolated from the spleens of infected mice at the indicated time-points post infection with LCMV docile.

All graphs show the mean and SEM. Each time-point represents combined data from 2-4 independent experiments (*n*=6-16 per time-point). Some error bars are not visible due to overlapping symbols. B cell (CD19^+^), cDC, conventional dendritic cell (CD11c^+^ MHC-II^+^), Granulocyte (CD11b^+^ Gr-1^Hi^), macrophage (CD11b^+^ Gr-1^Int^), * *p* < 0.05 (unpaired *t-*test).

**Supplementary Figure 4.**

**Supplementary Figure 4.**

Thioglycollate induced inflammatory macrophages were isolated from the abdominal cavities of naïve WT or *Ripk3^-/-^* mice, placed into culture and were left uninfected or were infected with LCMV docile at an MOI of 1.

**(A)** The proportion of WT or *Ripk3^-/-^* macrophages infected with LCMV (+LCMV), or left uninfected (-LCMV), were analysed by flow cytometry at 24 hours post infection.

**(B)** Viability of LCMV infected macrophages were determined by propidium iodide uptake (*n* = 4).

Data is expressed as fold change compared to uninfected. The mean and SEM are indicated.
